# Serum Amino Acid Profiling in Children with Autistic Spectrum Disorder: Insights from a Single-Center Study in Southern Romania

**DOI:** 10.3390/healthcare11182487

**Published:** 2023-09-07

**Authors:** Cătălina Mihaela Anastasescu, Veronica Gheorman, Florica Popescu, Eugen-Cristi Stoicănescu, Victor Gheorman, Anca-Lelia Riza, Oana Badea, Ioana Streață, Felicia Militaru, Ion Udriștoiu

**Affiliations:** 1 Hospital of Neuropsychiatry Craiova, Children Mental Health Center, Pharmacology Department, University of Medicine and Pharmacy of Craiova, 200349 Craiova, Romania; 2 Department of Cardiology, University of Medicine and Pharmacy of Craiova, 200349 Craiova, Romania; 3 Pharmacology Department, University of Medicine and Pharmacy of Craiova, 200349 Craiova, Romania; prof_floricapopescu@hotmail.com; 4 Pediatry Department, Emergency Clinical Hospital Râmnicu Vâlcea, 200300 Râmnicu Vâlcea, Romania; prof.dr.stoicanescu@gmail.com; 5 Department of Psychiatry, University of Medicine and Pharmacy of Craiova, 200349 Craiova, Romania; victor.gheorman@umfcv.ro (V.G.); feliciobanu@yahoo.com (F.M.); ion.udristoiu@umfcv.ro (I.U.); 6 Human Genomics Laboratory, University of Medicine and Pharmacy, 050474 Craiova, Romania; anca.costache@umfcv.ro (A.-L.R.); ioana.streata@umfcv.ro (I.S.); 7 Department of Modern Languages, University of Medicine and Pharmacy of Craiova, 200349 Craiova, Romania; o_voiculescu@yahoo.com

**Keywords:** amino acids, serum, autism spectrum disorder, biomarkers

## Abstract

The objective of this study was to analyze the serum amino acid profile in children diagnosed with autistic spectrum disorder (ASD) in southern Romania. The analysis aimed to provide insights into the underlying metabolic dysregulations associated with ASD. ASD is a neurodevelopmental disorder characterized by impaired social interaction, communication deficits, and restricted repetitive behaviors. Although the exact cause of ASD is largely unknown, recent evidence suggests that abnormalities in amino acid metabolism may contribute to its pathogenesis. Therefore, studying the amino acid profile in children with ASD could offer valuable information for understanding the metabolic disturbances associated with this complex disorder. This single-center study examined serum samples from children diagnosed with ASD, utilizing advanced analytical techniques to quantify the levels of different amino acids, amino acid derivatives, and amino acid-like substances. The results showed a lower level of taurine and a higher level of asparagine and leucine in the ASD group versus the control group. In the ASD group, we observed significant differences in tryptophan and alpha-aminobutyric acid levels based on age, with higher tryptophan levels in children older than 7 years when compared to children younger than 7 years; however, no significant correlations were found with the ASD group older than 7 years old. Additionally, younger children with ASD exhibited higher levels of alpha-aminobutyric acid than older children with ASD. The findings from this study contribute to the growing body of knowledge on the metabolic aspects of ASD, highlighting potential biomarkers and therapeutic targets for improving the management and treatment of ASD in children.

## 1. Introduction

In recent years, there has been a significant increase in the prevalence of autistic spectrum disorder (ASD). This has led to a growing need for early diagnosis and intervention. According to a report by the Centers for Disease Control and Prevention (CDC) in May 2023, approximately 1 in 36 children received a diagnosis of ASD [1]. ASD is characterized by difficulties in communication and social interactions, as well as restrictive and repetitive patterns of behavior and activities. These symptoms manifest during early development and can be diagnosed within the first 5 years of life [2]. The etiology of ASD is complex and multifactorial, involving a combination of genetic and environmental factors. Extensive research has been conducted in various domains, including anatomy, genetics, electroencephalography, imaging, immunology, metabolomics, and metallomics, to understand the underlying mechanisms of ASD [3,4,5]. Despite these efforts, there is currently no specific biomarker that can be used in clinical practice for the diagnosis of ASD due to its heterogeneity [6]. However, identifying more homogeneous groups of individuals with similar traits may lead to more effective approaches and outcomes in ASD management [7]. Emerging evidence suggests that the metabolism of amino acids may play a role in influencing the severity of ASD symptoms [5,8,9,10]. Amino acids, specifically those acting as neurotransmitters in the central nervous system (CNS), have been implicated in the pathophysiology of ASD [11,12,13]. For instance, certain excitatory amino acids, such as glutamate, can modulate cognitive functions like memory and learning, which are commonly impaired in individuals with ASD [11,14,15]. These findings have prompted researchers to investigate the potential utility of serum amino acid profiling as a diagnostic tool and to assess its relationship with ASD severity.

In light of emerging evidence linking amino acids to the development of autism spectrum disorder (ASD), we conducted a comprehensive study involving a group of autistic children from the southern region of Romania and compared it to a carefully selected control group. Our main goal was to evaluate the levels of specific amino acids in the serum of children with ASD in comparison to those without any psychiatric disorders. Through this meticulous investigation, our primary aim was to enhance our understanding of the causes of ASD and contribute valuable insights into the existing knowledge of autism biomarkers. Moreover, studying a geographically specific population may provide valuable insights into the potential influence of genetic and environmental factors on the etiology of ASD. Overall, our study aims to fill gaps in the existing literature and advance our understanding of the complex nature of ASD.

## 2. Materials and Methods

### 2.1. Informed Consent

The protocol of this study was approved by the University and Scientific Ethics and Deontology Commission of the University of Medicine and Pharmacy of Craiova (No. 154/24.09.2021), ensuring compliance with the Declaration of Helsinki and the Code of University Ethics. This confirmed that the research process complied with proper conduct, together with the codes of practice established by the Code of Medical Ethics. Informed consent was obtained from all parents or legal guardians to conduct the study and publish the resulting data. A contract was also signed with a local certified laboratory for the collection of the necessary samples and the laboratory determination of amino acid levels.

### 2.2. Study Groups

**Figure 1:**
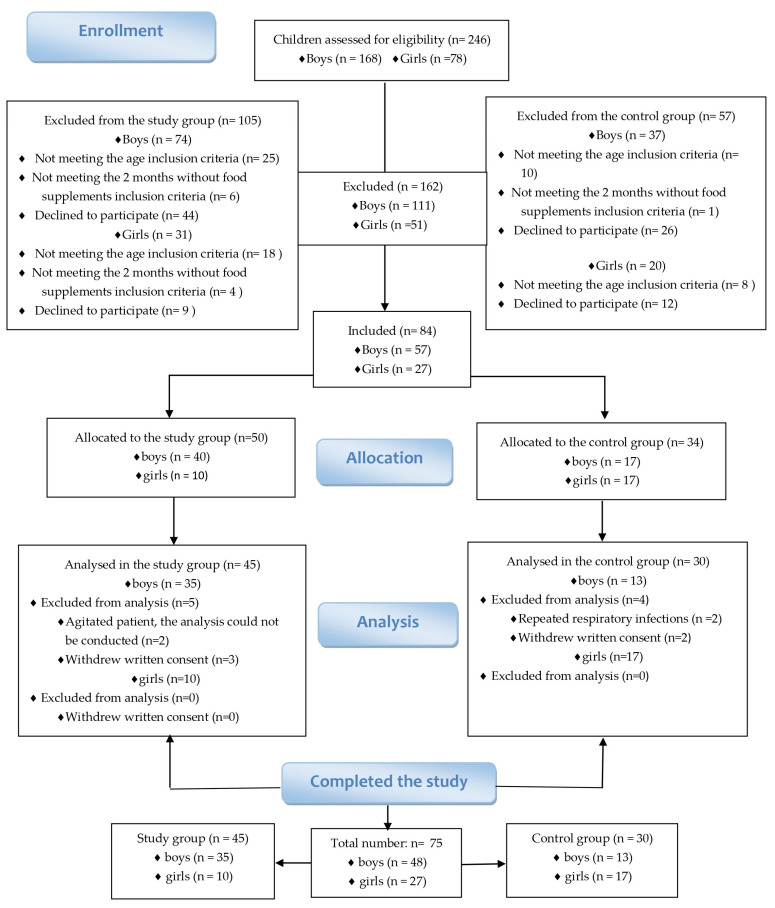
Consort Flow Diagram Illustrating the Enrollment of Children (Boys and Girls) in the Study Group and the Control Group.

This case–control study was conducted over a period of one year, from 2021 to 2022, among a cohort of 75 Romanian children aged between 2 and 12 years old. Both boys and girls were included in this study. Specifically, 45 children diagnosed with autism spectrum disorder (ASD) were selected as the study group, while the control group consisted of 30 children who did not have any psychiatric pathologies (Figure 1).

The participants for the study group were recruited from individuals who sought evaluation and diagnosis for ASD between 2017 and 2022 at a privately owned medical practice in Craiova, Romania. The control group was carefully chosen to match the ASD group in terms of age, sex, and other relevant demographic variables. Controls were recruited from local kindergartens, schools, and various community resources within the same geographic area as the ASD group.

The selection criteria were determined based on the population of interest and include children aged 2–12 years diagnosed with autistic spectrum disorder (ASD) according to established diagnostic criteria, such as the DSM-5, children with ASD who have not received any previous treatment or interventions that may potentially influence their serum amino acid profile, children who have not taken dietary supplements in the last 2 months prior to this study, children with ASD without any known underlying metabolic disorders or genetic conditions that may affect amino acid metabolism, and children whose parents or guardians provided informed consent for participation in this study.

Exclusion criteria include children with known genetic or metabolic disorders that could impact amino acid metabolism, children with significant medical conditions that may confound the results, and children taking medications or supplements known to affect amino acid levels.

### 2.3. Screening

To support the diagnosis of ASD, we used the Social Communication Questionnaire (SCQ). The SCQ is a widely used instrument that assesses social communication skills and features associated with autism spectrum disorder (ASD) [16]. The SCQ is a screening tool designed to identify individuals who may be at risk for ASD and can be used to support the diagnostic process. SCQ consists of 40 items that capture various social communication skills and behaviors commonly seen in individuals with ASD. These items cover areas such as reciprocal social interaction, communication, and repetitive or restricted patterns of behavior. Each item in the SCQ is rated as either “0” or “1”, indicating the presence or absence of the behavior or skill being assessed. Higher scores on the SCQ indicate a higher likelihood of ASD-related impairments [16]. The version of the SCQ we used has a cut-off score of 15, where scores above this threshold suggest a higher risk for ASD.

The SCQ is not intended to provide a definitive diagnosis of ASD, but rather serve as a screening tool to determine the need for further evaluation. It is often used in conjunction with other diagnostic assessments, such as clinical interviews and observations, to inform the diagnostic process [16]. It is important to note that, while the SCQ is a well-established tool, it is not without limitations. It relies on caregiver or informant reports and may be subject to biases or inaccuracies. Additionally, it should not be used as the sole basis for diagnosing ASD, but rather as part of a comprehensive evaluation.

The SCQ assessments were administered by a qualified child psychiatrist.

### 2.4. Collection and Testing of Laboratory Samples

The samples were collected between October 2021 and January 2022 at the Bioclinica Laboratory Collection Centre in Craiova, Romania. Patients and volunteers presented themselves in the early morning after fasting overnight, on a designated date.

The samples of venous blood were collected from each study participant in vacutainers with EDTA K3 to as much as the vacuum allowed. The plasma is separated, transferred into a plastic tube, and frozen immediately. The stability of the sample is 30 days at −20 °C. Due to the unavailability of local analysis, the collected samples were transported on dry ice under optimal transport conditions to the Reference Laboratory Metabolics, a medical laboratory located in L’Hospitalet de Llobregat, Barcelona, Spain. This laboratory possesses quality system certifications (ER-1087/1998) based on the UNE-EN ISO 9001 standard and environmental management system certifications (GA-2001/0146) based on the EN ISO 14001 standard, issued by AENOR.

To analyze the amino acid profile in the serum, we utilized liquid chromatography with tandem mass spectrometry (LC-MS/MS). LC-MS/MS is a sophisticated analytical technique that combines the separation capabilities of liquid chromatography with the high sensitivity and selectivity of triple quadrupole mass spectroscopy analysis. In contrast to traditional methods, LC-MS/MS offers user-friendly operation and a wide range of applications. For the analysis, a minimum volume of 1 mL of venous blood serum was necessary to obtain accurate results for each sample.

The results of the analysis were received within a timeframe of 12 to 15 working days following sample collection.

The analysis focused on a range of amino acids, including both basic amino acids and derivatives: alanine, alpha-aminobutyric acid, alpha-amino adipic acid, anserine, arginine, aspartic acid, asparagine, beta-alanine, citrulline, cystathionine, cysteine, glutamic acid, glutamine, glycine, histidine, homocysteine, hydroxylysine, hydroxyproline, isoleucine, leucine, lysine, methionine, methionine sulfoxide, methylhistidine, ornithine, phenylalanine, proline, sarcosine, serine, taurine, threonine, tryptophan, tyrosine, and valine. While basic amino acids play a crucial role in protein synthesis and various cellular functions, their derivatives offer additional insights into metabolic pathways and processes that may be altered in individuals with autism spectrum disorder (ASD). Amino acid derivatives can result from the metabolism of their respective amino acids and may serve as indicators of specific metabolic dysfunctions or imbalances. By including amino acid derivatives in our analysis, we aimed to capture a more comprehensive picture of amino acid metabolism in individuals with ASD and explore potential metabolic biomarkers or pathways that may be implicated in the development or etiology of ASD.

### 2.5. Statistical Analysis

The statistical analysis was conducted using IBM SPSS version 23. Graphs and charts were created using MS Office Excel 2016 using a Windows 10 operating system.

In order to examine the data, various statistical tests and visualizations were utilized. The Shapiro–Wilk and Kolmogorov–Smirnov tests were employed to assess the distribution of the data, while QQ plots and boxplots were utilized for visual examination.

For the comparative analysis, a Student’s *t*-test with a significance threshold of 0.05 was performed on groups with approximately Gaussian distributions. When multiple comparisons were necessary, the ANOVA test was employed with a significance threshold of 0.05. In cases where the coefficients of variation were equal, the Bonferroni correction was applied. On the other hand, Tamhane’s test was used when the variations were unequal.

For groups that did not follow a Gaussian distribution, the Mann–Whitney U test was employed for comparative analysis of the groups. When multiple comparisons were necessary, the Kruskal–Wallis test was utilized.

The statistical analysis of the two groups was conducted in four stages. Firstly, centroids were established based on age. Secondly, this study established a group centered around the age of 4 and another group centered around the age of 8. In the third stage, a discriminant analysis of clusters was performed to validate the groups. The ANOVA indicated that the two groups were statistically different (*p* < 0.0001). Wilks’ lambda suggested that the groups had relatively higher homogeneity, although a slight difference favored the study group. Finally, in the last stage called cross-validation, one patient under the age of 7 was identified, which resulted in a 4% error rate for the under 7 years of age group. Taking into account these findings, the age of 7 years was established as the reference age.

## 3. Results

### 3.1. Study Cohort: Individuals with Autism Spectrum Disorder (ASD) and Control Group Descriptive Data

#### 3.1.1. Gender and Age Group Distribution

The gender distribution within the study group and the control group was as follows: Within the study group, 35 boys and 10 girls were included, maintaining a reported ratio of 4:1, as cited in the literature. The control group consisted of 13 boys and 17 girls (Table 1).

Regarding the age group distribution, the study group comprised 25 children aged between 2 and 6 years and 20 children aged between 7 and 12 years. In the control group, there were 15 children aged 2–6 years and 15 children aged 7–12 years (Table 1).

#### 3.1.2. Environmental Distribution

The distribution of environmental backgrounds for both groups is outlined as follows: In the study group, 35 children hailed from urban environments while 10 children originated from rural areas. Within the control group, 26 children lived in urban environments and 4 children resided in rural locations (Table 1).

#### 3.1.3. Dietary Patterns and Nutritional Status

Dietary preferences within the study group and the control group exhibited the subsequent distribution: Within the study group, 35 children adhered to a restrictive diet, whereas 10 children maintained a diversified diet. In contrast, all children within the control group followed a diversified diet. Concerning the nutritional status of participants, the study group consisted of 18 underweight children, 20 with normal weight, 5 overweight, and 2 obese children. Within the control group, 6 children were classified as underweight, 23 had a normal weight, and 2 were categorized as overweight (Table 1).

### 3.2. The Results for the Screening Questionnaire Social Communication Questionnaire (SCQ)

The results of the screening questionnaire SCQ revealed an average score of 18.60, accompanied by a standard deviation of 3.74. We conducted a descriptive analysis of the three domains within the SCQ and assessed the normality hypothesis using the Lilliefors significance correction based on the Kolmogorov–Smirnov test. The resulting outcomes are as follows: For the social interaction domain of the SCQ, we obtained an average score of 6.73 ± 2.81, with a *p*-value of 0.052. The communication domain exhibited an average score of 5.78 ± 1.78, yielding a *p*-value of 0.000, while the domain related to restrictive and repetitive behavioral patterns displayed an average score of 5.44 ± 1.68, with a *p*-value of 0.008 (see Table 2). According to our study, utilizing a cut-off score of 15, the elevated score of 18.60 observed in the study group indicates a significantly heightened risk for autism spectrum disorder (ASD) in these patients.

### 3.3. Results for the Serum Amino Acid Profile

#### 3.3.1. Analysis of Amino Acids from the Study Group in Comparison to the Control Group

We performed a descriptive statistical analysis of the serum amino acid profile, amino acid derivatives (taurine), and amino acid-like substance (ornithine), using the *t*-test for equal variances and for unequal variances when applicable (Table 3).

**Figure 2:**
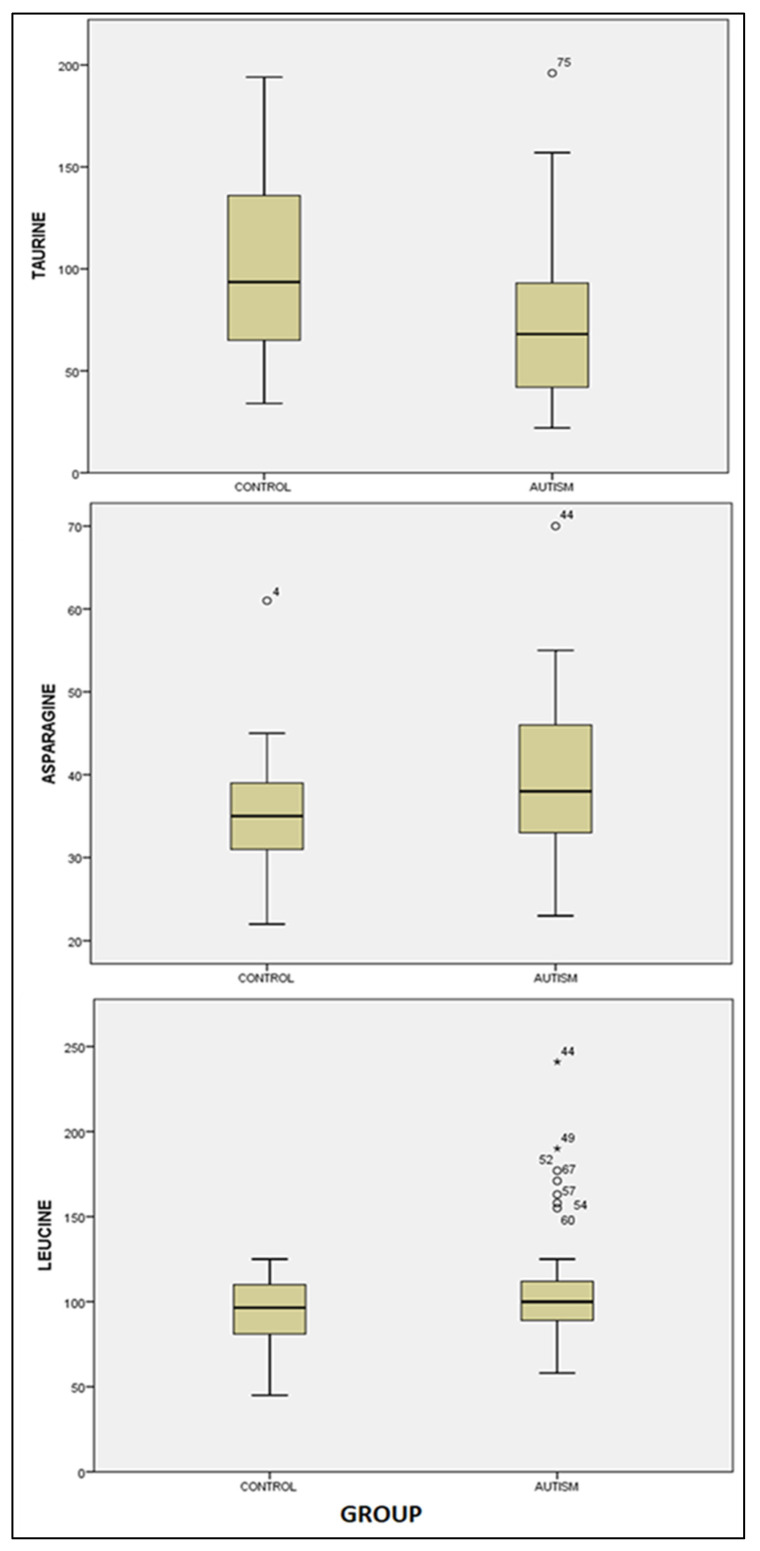
Boxplot graphs for taurine, asparagine and leucine levels in the study group (autism) versus the control group. The cases with extreme values outside the 95% CI (outliers) are represented by a circle and asterisk.

We identified noteworthy distinctions between the study group (consisting of children diagnosed with ASD) and the control group (comprising children without psychiatric pathology) solely in the cases of taurine, asparagine, and leucine. Specifically, for taurine, the mean concentration was 72.08 ± 37.75 μmol/L in the study group, in contrast to 100.16 ± 45.27 μmol/L in the control group. The concentration exhibited a significant reduction in the study group compared to the control group: t (73) = 2.912, *p* = 0.005. Concerning asparagine, we observed substantial differences: 39.75 ± 9.08 μmol/L in the study group versus 35.40 ± 7.10 μmol/L. Children with autism displayed significantly elevated levels of asparagine compared to their counterparts without psychiatric pathology: t = −2.323, *p* = 0.023. Moreover, we encountered significantly amplified values within the autism spectrum disorder group for leucine: 108.71 ± 35.21 μmol/L, in comparison to the control group’s value of 94.40 ± 19.20 μmol/L, t = −2.267, *p* = 0.026 (refer to Table 3 and Figure 2).

For alanine, alpha-aminobutyric acid, beta-alanine, glutamic acid, methylhistidine, sarcosine, and tryptophan, due to the non-parametric distribution of values in the two groups, we employed the Mann–Whitney U test to compare mean differences. However, our analysis did not yield significant results: alpha-amino adipic acid had a *p*-value of 0.730, Z = −0.345; alanine had a *p*-value of 0.825, Z = −0.222; beta-alanine had a *p*-value of 0.762, Z = −0.303; glutamic acid had a *p*-value of 0.721, Z = −0.358; methylhistidine had a *p*-value of 0.688, Z = −0.400; sarcosine had a *p*-value of 1.000, Z = 0.000; and tryptophan had a *p*-value of 0.983, Z = −0.022.

However, hydroxyzine, anserine, cystathionine, homocysteine, and methionine sulfoxide could not undergo statistical analysis, as the values obtained for all patients were unquantifiable, being less than 1 μmol/L.

#### 3.3.2. Analysis of Amino Acids Based on Gender in the Study Group Compared to the Control Group

Subsequently, we conducted a statistical analysis of the amino acid values categorized by male and female sex, as presented in Table 4. To interpret the results, we employed ANOVA for unequal variances. Despite observing that values for various amino acids within the study group were higher in boys compared to girls—namely, asparagine, citrulline, glutamine, glycine, histidine, hydroxyproline, isoleucine, leucine, lysine, methionine, methylhistidine, ornithine, phenylalanine, proline, serine, taurine, threonine, tyrosine, and valine—while values for alpha-aminobutyric acid, arginine, aspartic acid, and cysteine were lower, we did not identify statistically significant outcomes.

Notably, the mean values for boys within the study group were lower than those of boys in the control group, albeit lacking statistical significance. Similarly, we noted reduced values for girls in the study group, yet a statistical significance remained elusive. However, an exception emerged for taurine, where we observed significance between girls in the autism group and boys in the control group, with a *p*-value of 0.034. Nonetheless, we surmised that this finding might be a false positive due to sample size discrepancies (see Table 4).

Furthermore, as normal distribution assumptions were not met for alpha-amino adipic acid, alanine, beta-alanine, glutamic acid, sarcosine, and tryptophan among the groups, we employed the non-parametric Kruskal–Wallis test for conducting multiple comparisons. The results yielded *p*-values of 0.416 for alpha-amino adipic acid, 0.938 for alanine, 0.244 for beta-alanine, 0.936 for glutamic acid, 0.885 for sarcosine, and 0.401 for tryptophan.

#### 3.3.3. Statistical Analysis of Amino Acid Concentrations According to Age Groups

To perform the statistical analysis of amino acid concentrations according to age groups, we applied the ANOVA and Kruskal–Wallis tests for multiple comparisons and we obtained significant results for alpha-aminobutyric acid and asparagine (Table 5).

**Figure 3:**
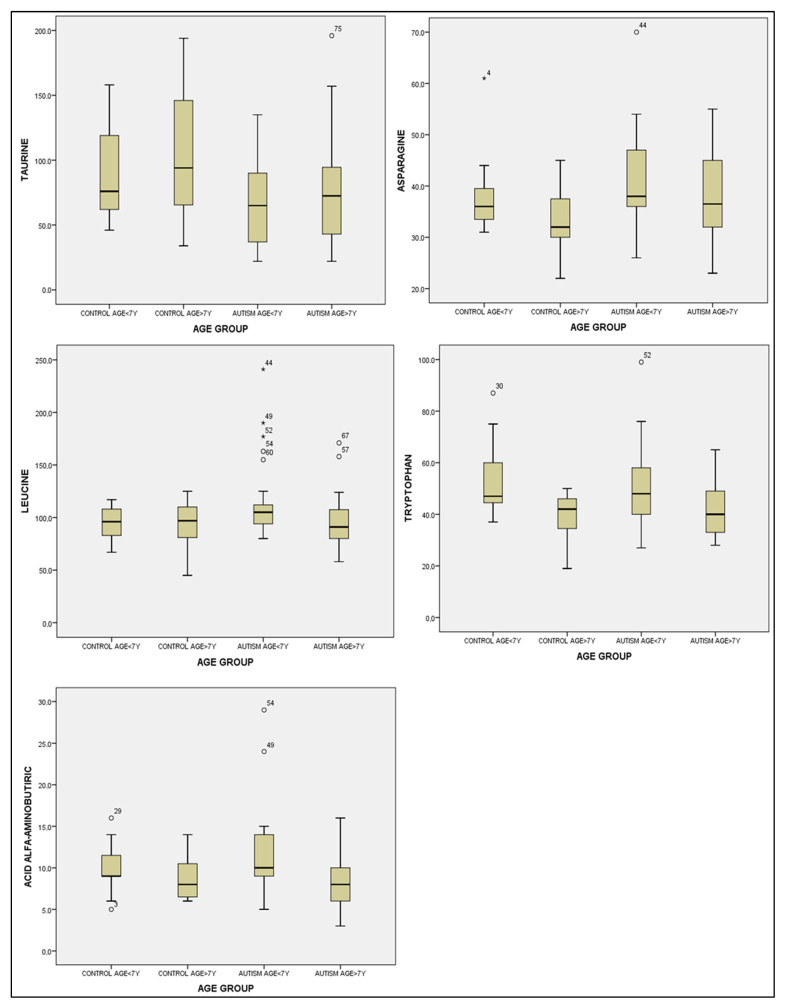
Boxplot graphs for asparagine, taurine, leucine, tryptophan and alpha-aminobutyric acid levels according to age: <7 years old and >7 years old for the control group and study (autism) group. The cases with extreme values outside the 95% CI (outliers) are represented by a circle and asterisk.

In regard to the taurine results across different age groups, we identified significant differences through the ANOVA test among the groups based on age, with an F-value of 3.518 and a *p*-value of 0.019. Similarly, for asparagine, significant differences emerged in the ANOVA test between age groups, where the F-value was 2.780 and the *p*-value was 0.047. Subsequently, a descriptive analysis was carried out with a Bonferroni correction for multiple comparisons of taurine and asparagine. Although statistically significant correlations were observed between children in the control group over 7 years of age and children in the study group under 7 years of age (*p* = 0.17 for taurine and *p* = 0.032 for asparagine), these findings were not taken into consideration. It is plausible that the limited number of children in the study groups might have contributed to this outcome (refer to Table 6 and Figure 3).

In the case of leucine, a Kruskal–Wallis analysis was conducted for multiple comparisons across age groups, yet no statistically significant correlations were identified, with a *p*-value of 0.149 (see Figure 3).

With respect to the results obtained for tryptophan, significant differences were found through the ANOVA test for the age groups, yielding a *p*-value of 0.006. Similarly, for alpha-aminobutyric acid, significant differences surfaced in the ANOVA test among the age groups, resulting in a *p*-value of 0.019.

Following these findings, a descriptive analysis based on age groups was conducted with a Bonferroni correction. Notably, significantly higher levels of tryptophan were detected in children from the control group above 7 years of age: 39.13 ± 9.21 μmol/L, as compared to children from the control group below 7 years of age: 5.53 ± 13.59 μmol/L, with a *p*-value of 0.024. Furthermore, a significant difference was observed when comparing children from the control group above 7 years to those from the study group below 7 years: 51.04 ± 16.23 μmol/L, with a *p*-value of 0.046. For alpha-aminobutyric acid, substantially higher values were evident in children from the study group under 7 years: 11.76 ± 5.40 μmol/L, as opposed to children from the study group over 7 years: 8.15 ± 3.21 μmol/L, with a *p*-value of 0.02 (see Table 6 and Figure 3).

## 4. Discussion

We conducted an analysis of 34 amino acids in the serum of children diagnosed with autism spectrum disorder (ASD) and compared them to a control group of children. Our investigation revealed significant differences in the levels of taurine, asparagine, and leucine between the two groups [1].

### 4.1. Taurine’s Potential Role in Neurodevelopment

This observation aligns with the growing body of literature highlighting the potential role of taurine in neurodevelopmental disorders. Taurine has been shown to have a vital role in various developmental processes, including eye and brain development, as well as reproductive function [17,18]. Moreover, studies on laboratory animals have indicated associations between low taurine levels and pathologies such as cardiomyopathies, retinal degeneration, and growth retardation, particularly during developmental periods [19,20,21].

### 4.2. Gender and Amino Acid Levels

In a study conducted by Bugajska in 2017, lower levels of citrulline, aminobutyric acid, isoleucine, leucine, tryptophan, and ornithine were reported in groups of autistic boys compared to healthy boys [22]. However, in our study, we did not find any statistically significant differences between male and female participants. While arginine, cystine, alpha-aminobutyric acid, and aspartic acid levels were lower in boys compared to girls, the differences were not statistically significant. The boys in the study group had a lower mean value compared to the boys in the control group, but again, this difference was not statistically significant. Similarly, the girls in the study group had lower values, but this was also not statistically significant. It is possible that these non-significant findings are due to the lack of homogeneity in the study groups [22].

### 4.3. Age-Related Insights

Regarding age, we noted significant values for tryptophan and alpha-aminobutyric acid. Specifically, we found higher tryptophan levels in children older than 7 years in the control group compared to children younger than 7 years in both the autism spectrum disorder (ASD) group and the study group. Our study’s findings, however, contradicted previous studies that suggested lower tryptophan levels in children with ASD [23,24]. Notably, both children with ASD and those in the control group younger than 7 years had lower tryptophan levels compared to older children. This suggests that tryptophan, as a precursor of serotonin, may influence the expression and severity of autism by affecting the developmental process [25].

### 4.4. Alpha-Aminobutyric Acid and Dietary Influences

The levels of alpha-aminobutyric acid were found to be significantly higher across the different groups according to the ANOVA test (F(3) = 3.517; *p* = 0.019). Specifically, the group of children with autism under the age of 7 had higher concentrations compared to the group of children with autism who were above the age of 7, with a mean difference of 3.620 (*p* = 0.020). Changes in the values of these amino acids may be due to poor nutrition or the specific food preferences of children with autism [26]. In our study, a substantial majority (77.77%) of the children with ASD had a restricted diet or specific food preferences. However, the results indicated that only a small portion of the children with autism had amino acid deficiencies due to their restrictive diet. Therefore, while the dietary patterns of children with autism may not necessarily lead to amino acid deficiencies, they may contribute to certain imbalances in their amino acid levels, which in turn could be associated with the manifestation of symptoms [4].

### 4.5. Taurine and Amino Acid Imbalances

Several studies have found a potential correlation between taurine levels and symptoms of autism spectrum disorder (ASD), suggesting that taurine could be a biomarker in specific ASD subgroups [27]. Another study observed high levels of glutamic acid and aspartic acid, as well as low levels of phenylalanine, tryptophan, methionine, and tyrosine in a group of children with ASD [23]. Additionally, increased levels of tryptophan were found to be associated with ASD symptoms. Researchers also noted that children with ASD had elevated levels of neuroactive amino acids and decreased levels of essential amino acids [9]. Specifically, lower levels of plasma cysteine, tyrosine, and serine, as well as higher levels of glutamic acid, were observed in autistic children compared to a control group. This study also reported lower levels of α-amino adipic acid, carnosine, and β-alanine, and higher levels of hydroxyproline, phosphoserine, and β-aminoisobutyric acid in autistic children [25]. Another study showed that increased plasma levels of glutamate were associated with the pathogenesis of ASD [11]. Furthermore, glutamate and homocysteine were found to be increased in autistic children, while glutamine and tryptophan levels were decreased. The findings regarding the plasma levels of taurine and lysine remain controversial [28]. In another study examining the metabolic aspects of ASD, higher levels of glutamic acid, phenylalanine, asparagine, tyrosine, alanine, and lysine were observed compared to controls, along with reduced plasma glutamine [8]. A large case–control study also revealed that glutamine, glycine, and ornithine were altered in children with ASD, highlighting the potential significance of amino acid metabolism in the disorder [3].

### 4.6. Asparagine and Future Directions

In our study, a significant difference was observed regarding asparagine levels between the two groups. Specifically, we found that children with autism had significantly higher levels of asparagine compared to the control group. However, there is limited research examining the role of asparagine in autism, so further investigation is needed. Asparagine is classified as a non-essential amino acid, meaning it can be converted into aspartate to enhance cellular functions. It also plays a role in cellular exchanges, particularly with serine, histidine, and arginine, which contribute to cell proliferation. Additionally, asparagine has a similar affinity to transporter proteins as glutamine [29,30]. It is possible that the elevated levels of asparagine observed in the autism group could be attributed to a deficiency in glutamine [23]. However, we did not examine this hypothesis in our study, and we did not find any significant differences in glutamine levels between the groups. To strengthen our findings, future studies should explore the potential relationship between asparagine, glutamine, and autism, as well as investigate the implications of elevated asparagine levels on cellular functions and overall health in individuals with autism.

## 5. Limitations of this Study

This study is not without its limitations, which should be acknowledged when interpreting the results. The sample size of this study was relatively small, encompassing 75 Romanian children, with 45 children in the study group and 30 children in the control group. A larger and more diverse sample size would have enhanced the robustness and generalizability of the findings. Additionally, a potential selection bias could have been introduced, as participants for the study group were recruited from individuals seeking evaluation and diagnosis for autism spectrum disorder (ASD) at a medical practice. This may not fully represent the entirety of children with ASD in the general population, some of whom might not seek medical evaluation. The gender distribution within both groups was imbalanced, and variations in dietary habits between the groups could have contributed to observed differences in serum amino acid profiles. These factors should be considered when interpreting this study’s outcomes. Future research should strive for a more representative sample, balanced gender distribution, and controlled dietary factors to minimize potential confounding effects.

Furthermore, this study’s design lacked a longitudinal follow-up, preventing insights into the intervention’s long-term effects. This study also did not adequately control for potential confounding variables such as socioeconomic status, parental educational background, and home support, which could influence the outcomes. Blinding procedures were not implemented, potentially introducing bias in outcome assessment and reporting. Moreover, this study’s single-center nature in southern Romania raises questions about the generalizability of the findings to different populations and settings. Cultural, genetic, and environmental factors specific to the region might impact the results, limiting their applicability to a broader context.

## 6. Specific Next Steps

To address these limitations and further advance the understanding of the intervention’s impact on autism spectrum disorder (ASD), several specific next steps are recommended. Replicating this study with a larger and more diverse sample size would bolster statistical power and increase the external validity of the findings. Collaborating with multiple research centers across different regions or countries through a multi-center study would enhance the generalizability of the results. A longitudinal follow-up study is crucial to uncover the intervention’s long-term effects and sustainability. Implementing a randomized control trial design with proper randomization and blinding procedures would enhance this study’s validity and reliability. Comparative studies involving different interventions or treatment approaches would provide valuable insights into the relative effectiveness of the studied intervention. Incorporating a comprehensive array of assessment measures would yield a more holistic understanding of the intervention’s impact on various outcomes. By undertaking these steps, the research community can further elucidate the intervention’s potential and contribute to refining diagnostic and therapeutic strategies for individuals with ASD.

## 7. Conclusions

Our study underscores the significant role of amino acids in autism spectrum disorder (ASD), highlighting potential links between disrupted amino acid metabolism and the expression of ASD symptoms. Variations in serum amino acid profiles were observed between children with ASD and those without psychiatric disorders, with specific amino acids like taurine, asparagine, leucine, tryptophan, and alpha-aminobutyric acid displaying notable differences. Age also appeared to influence amino acid levels, particularly in younger children. While this study suggests the possibility of amino acid profiles serving as clinical assessment tools for ASD, further research with larger, better-matched cohorts and controlled variables is essential for robust conclusions. Moreover, investigating genetic and environmental factors contributing to altered amino acid metabolism could potentially lead to novel therapeutic strategies for ASD. Overall, this study contributes to the growing understanding of amino acids’ roles in ASD but highlights the need for continued investigations to establish concrete biomarkers and effective interventions.

## Figures and Tables

**Table 1:** Descriptive data of the study group and the control group.

Demographic Data Distribution		Study Group	Control Group
Gender distribution	Boys	35	13
	Girls	10	17
Age group distribution	2–6 years	25	15
	7–12 years	20	15
Environment distribution	Urban	35	26
	Rural	10	4
Dietary types distribution	Restrictive diet	35	0
	Diversified diet	10	30
The nutritional status	Underweight	18	6
	Normal weight	20	22
	Overweight	5	2
	Obese	2	0

**Table 2:** Descriptive analysis for SCQ domains in the Study Group.

SCQ Domains	Mean Score	Standard Deviation	*p*-Value (Kolmogorov—Smirnov Test *)
Social interaction	6.73	2.81	0.052
Communication	5.78	1.78	0.000
Restrictive and repetitive behavioral patterns	5.44	1.68	0.008

* *Lilliefors Significance Correction*.

**Table 3:** Distribution of amino acids, amino acid derivatives (taurine), and amino acid-like substance (ornithine) based on the mean concentration expressed in μmol/L and standard deviation and statistical differences between groups.

Amino Acids	Autism Group n = 45	Control Group n = 30	*t*-Test *	
	Mean ± Standard Deviation (μmol/L)	Mean ± Standard Deviation (μmol/L)	t-Value	*p*-Value
alpha-aminobutyric acid	10.15 ± 4.86	9.43 ± 2.83	0.810	0.420
arginine	67.73 ± 17.33	67.40 ± 17.16	−0.082	0.935
asparagine	39.75 ± 9.08	35.40 ± 7.10	−2.323	0.023
aspartic acid	10.42 ± 3.75	9.63 ± 2.80	−1.039	0.302
citrulline	34.64 ± 27.92	28.76 ± 8.80	−1.317	0.193
cysteine	17.20 ± 11.91	22.03 ± 13.52	−1.630	0.107
glycine	200.97 ± 41.03	195.90 ± 46.89	−0.496	0.622
glutamine	632.75 ± 136.09	675.46 ± 124.15	−1.404	0.165
histidine	68.06 ± 15.78	65.43 ± 15.44	−0.714	0.477
hydroxyproline	14.71 ± 5.84	14.96 ± 4.18	−0.221	0.826
isoleucine	54.911 ± 17.75	50.167 ± 9.68	−1.491	0.140
leucine	108.71 ± 35.21	94.40 ± 19.20	−2.267	0.026
lysine	132.84 ± 37.09	128.66 ± 30.65	−0.531	0.597
methionine	26.667 ± 9.38	24.800 ± 6.46	−1.019	0.311
ornithine	49.11 ± 20.10	52.00 ± 20.36	−0.600	0.546
phenylalanine	46.467 ± 13.15	42.433 ± 9.40	−1.547	0.126
proline	152.35 ± 60.85	147.66 ± 61.32	−0.325	0.746
serine	115.82 ± 31.55	103.80 ± 31.87	−1.607	0.113
taurine	72.08 ± 37.75	100.16 ± 45.27	−2.912	0.005
threonine	114.75 ± 39.21	104.30 ± 30.91	−1.287	0.202
tyrosine	58.867 ± 20.00	55.267 ± 16.72	−0.843	0.402
valine	195.33 ± 63.28	176.43 ± 48.35	−1.463	0.148

* Results of the *t*-test for equal and unequal variances, that shows statistical differences between groups, with a significance threshold of *p* < 0.05; n = number of children included in each group.

**Table 4:** Mean plasma amino acid concentrations expressed in μmol/L and standard deviation, according to male and female sex for the autism group and control group.

Amino Acids	Autism Group n = 45 Mean ± Standard Deviation		Control Group n = 30 Mean ± Standard Deviation		*p*-Value
	Male (n = 35)	Female (n = 10)	Male (n = 13)	Female (n = 17)	
alpha-aminobutyric acid	9.88 ± 4.49	11.10 ± 6.17	8.46 ± 2.50	10.17 ± 2.92	0.492
arginine	64.42 ± 17.15	65.30 ± 18.64	69.92 ± 17.12	65.47 ± 17.46	1
asparagine	39.77 ± 8.56	39.70 ± 9.26	35.15 ± 1.51	35.58 ± 8.29	1
aspartic acid	10.28 ± 3.41	10.90 ± 4.62	9.84 ± 2.85	9.47 ± 2.85	0.736
citrulline	36.31 ± 30.96	28.80 ± 11.89	29.61 ± 6.87	28.11 ± 10.20	0.551
cystine	15.77 ± 11.31	22.20 ± 13.20	24.07 ± 13.73	20.47 ± 13.56	1
glutamine	637.17 ± 139.81	617.30 ± 130.47	653.41 ± 96.86	692.41 ± 142.12	0.448
glycine	206.08 ± 42.37	183.10 ± 31.49	199.38 ± 55.22	193.23 ± 41.00	0.466
histidine	68.42 ± 15.10	66.80 ± 18.80	65.76 ± 12.63	65.17 ± 17.67	1
hydroxyproline	14.80 ± 6.13	14.40 ± 4.94	15.30 ± 3.90	14.70 ± 4.96	0.981
isoleucine	56.25 ± 18.22	50.20 ± 15.93	50.23 ± 6.27	50.11 ± 11.84	0.397
leucine	111.05 ± 35.90	100.50 ± 33.07	95.38 ± 15.81	93.64 ± 21.89	0.178
lisine	136.68 ± 38.91	119.40 ± 27.34	125.61 ± 31.75	131.00 ± 30.55	1
methionine	27.88 ± 10.04	22.40 ± 4.90	24.53 ± 5.37	25.00 ± 7.34	0.234
methylhistidine	2.22 ± 1.11	2.00 ± 1.76	2.23 ± 0.83	2.05 ± 0.89	1
ornithine	49.62 ± 19.35	47.30 ± 23.57	53.30 ± 26.34	51.00 ± 15.11	1
phenylalanine	47.05 ± 13.90	44.40 ± 10.50	42.84 ± 8.33	42.11 ± 10.39	0.486
proline	156.34 ± 65.93	138.40 ± 37.44	146.23 ± 46.68	148.76 ± 71.96	0.855
serine	117.34 ± 34.10	110.50 ± 20.79	105.84 ± 24.50	102.23 ± 37.22	0.400
taurine	75.51 ± 35.99	60.10 ± 43.21	109.15 ± 50.57	93.29 ± 41.00	0.034
threonina	119.60 ± 41.91	97.80 ± 21.64	96.46 ± 28.08	110.29 ± 32.45	0.145
tyrosine	60.94 ± 21.14	51.60 ± 13.82	51.84 ± 13.45	57.88 ± 18.82	0.344
valine	196.91 ± 62.49	189.80 ± 69.14	176.84 ± 29.27	176.11 ± 59.96	0.577

Results for ANOVA between groups with a significance threshold of *p* < 0.05; n = number of children included in each group.

**Table 5:** Mean amino acid concentrations in μmol/L according to age groups, <7 years and >7 years for the study group (autism) and <7 years and >7 years for the control group.

Amino Acids	Autism Group n = 45 Mean ± Standard Deviation		Control Group n = 30 Mean ± Standard Deviation			
	Age <7 Ani (n = 25)	Age >7 Ani (n = 20)	Age <7 Ani (n = 15)	Age >7 Ani (n = 15)	f(3)	*p*-Value
alpha-aminobutyric acid	11.76 ± 5.40	8.15 ± 3.21	10.06 ± 2.89	8.80 ± 2.73	3.517	0.019
asparagine	40.76 ± 9.54	38.50 ± 8.53	37.80 ± 7.55	33.00 ± 5.90	2.780	0.047
arginine	64.84 ± 17.08	71.35 ± 71.35	69.66 ± 17.72	65.66 ± 16.88	0.70	0.554
aspartic acid	11.40 ± 4.39	9.20 ± 2.33	10.40 ± 2.19	8.86 ± 3.20	5.86	0.119
alanine	286.04 ± 105.22	294.05 ± 76.80	294.20 ± 101.58	282.00 ± 117.69	0.62	0.980
betha-alanine	3.80 ± 1.35	3.20 ± 1.10	3.40 ± 0.91	3.40 ± 1.12	1.922	0.589
citrulline	39.68 ± 36.28	28.35 ± 8.27	32.13 ± 10.33	25.40 ± 5.43	1.624	0.191
cysteine	14.44 ± 10.32	20.65 ± 13.09	17.33 ± 12.77	26.73 ± 12.98	3.420	0.022
glutamic acid	21.72 ± 10.42	18.20 ± 7.14	27.86 ± 24.88	17.66 ± 8.05	1.87	0.142
glutamine	621.20 ± 165.67	647.200 ± 88.46	698.66 ± 140.66	652.26 ± 104.83	1.080	0.363
glycine	202.24 ± 45.98	199.40 ± 34.97	203.33 ± 37.54	188.46 ± 55.02	0.384	0.764
histidine	71.24 ± 18.34	64.10 ± 11.02	70.66 ± 14.66	60.20 ± 14.84	2.172	0.099
hydroxyproline	15.32 ± 6.22	13.95 ± 5.38	15.66 ± 4.16	14.26 ± 4.23	0.440	0.725
isoleucine	58.56 ± 20.02	50.35 ± 13.56	51.00 ± 8.06	49.33 ± 11.29	1.758	0.163
leucine	116.72 ± 38.95	98.70 ± 27.65	94.66 ± 16.19	94.13 ± 22.39	5.333	0.149
lysine	133.20 ± 44.07	132.40 ± 27.06	134.66 ± 27.41	122.66 ± 33.43	0.382	0.766
methionine	27.24 ± 10.60	25.95 ± 7.81	26.40 ± 6.62	23.20 ± 6.10	0.749	0.527
methylhystidine	2.32 ± 1.37	2.00 ± 1.12	1.80 ± 0.67	2.46 ± 0.91	4.007	0.261
ornithine	43.44 ± 17.81	56.20 ± 20.97	55.93 ± 19.90	48.06 ± 20.72	2.083	0.110
phenylalanine	49.48 ± 15.43	42.70 ± 8.56	43.46 ± 8.33	41.40 ± 10.56	3.967	0.265
proline	162.44 ± 62.61	139.75 ± 57.63	160.46 ± 75.60	134.86 ± 41.51	0.999	0.399
sarcosine	1.68 ± 0.69	1.20 ± 0.41	1.60 ± 0.73	1.33 ± 0.48	7.435	0.059
serine	118.64 ± 29.30	112.30 ± 34.60	110.20 ± 38.84	97.40 ± 22.54	1.414	0.246
threonine	117.36 ± 40.38	111.50 ± 38.47	108.93 ± 28.19	99.66 ± 33.74	0.751	0.525
taurine	67.12 ± 32.68	78.30 ± 43.32	91.86 ± 37.65	108.46 ± 51.77	3.518	0.019
tryptophan	51.04 ± 16.23	42.40 ± 11.22	53.53 ± 13.59	39.13 ± 9.21	4.542	0.006
tyrosine	57.84 ± 21.19	60.15 ± 18.86	59.60 ± 16.40	50.93 ± 16.43	0.807	0.494
valine	213.96 ± 71.41	172.05 ± 42.49	180.33 ± 52.13	172.53 ± 45.74	7.647	0.054

SD = Standard Deviation, n = number of subjects, *p* = result of the ANOVA test for multiple comparisons with significance threshold *p* < 0.05, f(3) = ANOVA ratio.

**Table 6:** Bonferroni correction for multiple comparisons according to age groups.

		Taurine				
(I) Age Group	(J) Age Group	Mean Difference (I-J)	Std. Error	Sig.	95% Confidence Interval	
					Lower Bound	Upper Bound
Control age < 7 years	Control age > 7 years	−16.600	14.930	1.000	−57.123	23.923
	Autism age < 7 years	24.746	13.354	0.408	−11.499	60.992
	Autism age > 7 years	13.566	13.965	1.000	−24.340	51.473
Control age > 7 years	Control age < 7 years	16.600	14.930	1.000	−23.923	57.123
	Autism age < 7 years	41.346 *	13.354	0.017	5.101	77.592
	Autism age > 7 years	30.166	13.965	0.205	−7.740	68.073
Autism age < 7 years	Control age < 7 years	−24.746	13.354	0.408	−60.992	11.499
	Control age > 7 years	−41.346 *	13.354	0.017	−77.592	−5.101
	Autism age > 7 years	−11.180	12.266	1.000	−44.473	22.113
Autism age > 7 years	Control age < 7 years	−13.566	13.965	1.000	−51.473	24.340
	Control age > 7 years	−30.166	13.965	0.205	−68.073	7.740
	Autism age < 7 years	11.180	12.266	1.000	−22.113	44.473
		Asparagine				
Control age < 7 years	Control age > 7 years	4.800	3.021	0.700	−3.401	13.001
	Autism age < 7 years	−2.960	2.702	1.000	−10.296	4.376
	Autism age > 7 years	−0.700	2.826	1.000	−8.372	6.972
Control age > 7 years	Control age < 7 years	−4.800	3.021	0.700	−13.001	3.401
	Autism age < 7 years	−7.760 *	2.702	0.032	−15.096	−.424
	Autism age > 7 years	−5.500	2.826	0.334	−13.172	2.172
Autism age < 7 years	Control age < 7 years	2.960	2.702	1.000	−4.376	10.296
	Control age > 7 years	7.760 *	2.702	0.032	0.424	15.096
	Autism age > 7 years	2.260	2.482	1.000	−4.478	8.998
Autism age > 7 years	Control age < 7 years	0.700	2.826	1.000	−6.972	8.372
	Control age > 7 years	5.500	2.826	.334	−2.172	13.172
	Autism age < 7 years	−2.260	2.482	1.000	−8.998	4.478
		Tryptophan				
Control age < 7 years	Control age > 7 years	14.400 *	4.844	0.024	1.251	27.549
	Autism age < 7 years	2.493	4.333	1.000	−9.267	14.254
	Autism age > 7 years	11.133	4.531	0.099	−1.166	23.433
Control age > 7 years	Control age < 7 years	−14.400 *	4.844	0.024	−27.549	−1.251
	Autism age < 7 years	−11.906 *	4.333	0.046	−23.667	−0.146
	Autism age > 7 years	−3.266	4.531	1.000	−15.566	9.033
Autism age < 7 years	Control age < 7 years	−2.493	4.333	1.000	−14.254	9.267
	Control age > 7 years	11.906 *	4.333	0.046	0.146	23.667
	Autism age > 7 years	8.640	3.980	0.200	−2.163	19.443
Autism age > 7 years	Control age < 7 years	−11.133	4.531	0.099	−23.433	1.166
	Control age > 7 years	3.266	4.531	1.000	−9.033	15.566
	Autism age < 7 years	−8.640	3.980	0.200	−19.443	2.163
		Alpha-amino	butiric	acid		
Control age < 7 years	Control age > 7 years	1.266	1.449	1.000	−2.667	5.200
	Autism age < 7 years	−1.693	1.296	1.000	−5.212	1.825
	Autism age > 7 years	1.916	1.355	0.971	−1.763	5.596
Control age > 7 years	Control age < 7 years	−1.266	1.449	1.000	−5.200	2.667
	Autism age < 7 years	−2.960	1.296	0.152	−6.478	0.558
	Autism age > 7 years	0.650	1.355	1.000	−3.030	4.330
Autism age < 7 years	Control age < 7 years	1.693	1.296	1.000	−1.825	5.212
	Control age > 7 years	2.960	1.296	0.152	−0.558	6.478
	Autism age > 7 years	3.610 *	1.190	0.020	0.378	6.842
Autism age > 7 years	Control age < 7 years	−1.916	1.355	0.971	−5.596	1.763
	Control age > 7 years	−0.650	1.355	1.000	−4.330	3.030
	Autism age < 7 years	−3.610 *	1.190	0.020	−6.842	−0.378

* The mean difference is significant at the 0.05 level.

## Data Availability

We confirm that the data supporting the findings of this study are available within the article.
